# Effects of isolation housing stress and mouse strain on intravenous cocaine self-administration, sensory stimulus self-administration, and reward preference

**DOI:** 10.1038/s41598-023-29579-9

**Published:** 2023-02-16

**Authors:** Michael Leonardo, Sarah Brunty, Jessica Huffman, Deranda B. Lester, Price E. Dickson

**Affiliations:** 1 Department of Biomedical Sciences, Joan C. Edwards School of Medicine, Marshall University, 1700 3rd Ave., Huntington, WV 25703 USA; 2 Department of Psychology, University of Memphis, 202 Psychology Building, Memphis, TN 38152 USA

**Keywords:** Behavioural genetics, Addiction

## Abstract

Sensory stimuli are natural rewards in mice and humans. Consequently, preference for a drug reward relative to a sensory reward may be an endophenotype of addiction vulnerability. In this study, we developed a novel behavioral assay to quantify the preference for intravenous drug self-administration relative to sensory stimulus self-administration. We used founder strains of the BXD recombinant inbred mouse panel (C57BL/6J, DBA/2J) and a model of stress (isolation vs enriched housing) to assess genetic and epigenetic effects. Following 10 weeks of differential housing, all mice were tested under three reward conditions: sensory rewards available, cocaine rewards available, both rewards available. When a single reward was available (sensory stimuli or cocaine; delivered using distinct levers), DBA/2J mice self-administered significantly more rewards than C57BL/6J mice. When both rewards were available, DBA/2J mice exhibited a significant preference for cocaine relative to sensory stimuli; in contrast, C57BL/6J mice exhibited no preference. Housing condition influenced sensory stimulus self-administration and strain-dependently influenced inactive lever pressing when both rewards were available. Collectively, these data reveal strain effects, housing effects, or both on reward self-administration and preference. Most importantly, this study reveals that genetic mechanisms underlying preference for a drug reward relative to a nondrug reward can be dissected using the full BXD panel.

## Introduction

Drug addiction, a devastating psychiatric disorder that manifests in a subset of drug users, is driven by highly heritable but unknown mechanisms^1^. Identifying the genetic drivers underlying the progression from controlled to compulsive drug use in vulnerable drug users is critical to reducing the morbidity, mortality, and societal costs resulting from drug addiction. In that regard, heritable variation in the motivation to seek out drug rewards rather than natural rewards may play a key role in addiction vulnerability. In humans and mice, complex sensory stimuli are reinforcing^2–6^ and are key components of many of the activities that humans find rewarding (e.g., sports, movies, music). Because sensory rewards provide an alternative to drug rewards, a strong preference for a drug infusion relative to a sensory stimulus may provide an endophenotype of addiction vulnerability, whereas a strong preference for a sensory stimulus relative to a drug infusion may provide an endophenotype of addiction resistance.

In two of our previous systems genetics studies, we separately indexed intravenous cocaine self-administration^7^ and self-administration of sensory stimuli^8^ in strains from the genetically complex BXD recombinant inbred mouse panel. Both phenotypes were heritable in BXD strains, and most mice rapidly acquired responding for intravenous cocaine and sensory stimuli. Separately, each of these approaches provides only a baseline index of reinforcement from a single reward rather than a preference for one reward relative to another. In contrast, integrating these techniques within the same operant conditioning paradigm could provide a readily tractable model for quantifying the preference for an intravenous drug reward relative to a sensory reward in the mouse. To our knowledge, this phenotype has never been quantified in mice although there is prior literature on cocaine choice involving other nondrug rewards in rats^9–12^. Developing a paradigm to quantify preference for a drug reward relative to a natural reward in distinct mouse strains would be the first step towards identification of genetic mechanisms underlying this phenotype which could be a critical vector driving drug addiction.

In the present study, we developed an operant conditioning paradigm to quantify the preference for cocaine relative to sensory stimuli in mice by integrating our previously described methods for quantification of intravenous cocaine self-administration^7^ and sensory stimulus self-administration^8^. Because our long-term goal is to identify genetic mechanisms underlying this phenotype, we developed our behavioral assay using two genetically distinct mouse strains: C57BL/6J and DBA/2J. We chose these strains because they are the founders of the BXD recombinant inbred panel which we^7,8,13–15^ and others^16–20^ have used in the context of a systems genetics approach to discover and characterize mechanisms underlying variation in behavioral and molecular phenotypes. Because our previous work has shown that environmental enrichment relative to isolation housing strain-dependently influences reinforcement from sensory stimuli and other addiction-relevant behaviors in BXD founders^6,21^, we included housing condition (isolated, enriched) as an independent variable. To eliminate fighting in group housed mice, we used female mice as experimental subjects.

Mice were housed in isolation or enrichment beginning at wean and continuing until the end of behavioral testing. Following 10 weeks of differential housing, mice were tested on an operant conditioning paradigm in which they could press a lever to self-administer a sensory stimulus using our previously described methods^5,6,8^. Mice were then surgically implanted with an intravenous jugular catheter under isoflurane anesthesia. Following surgical recovery, mice were tested in the same chamber using an intravenous drug self-administration paradigm in which they could self-administer cocaine using our previously described methods^7,22,23^. Importantly, the lever to self-administer sensory stimuli was distinct from the lever to intravenously self-administer cocaine. To quantify preference for cocaine relative to sensory stimuli, mice were tested on a stage during which both levers were available, and mice could self-administer either of the two rewards. These data were subsequently analyzed to determine the effects of strain and housing condition on intravenous self-administration of cocaine, self-administration of sensory stimuli, and the preference for an intravenous cocaine reward relative to a sensory reward when both rewards were available.

## Materials and methods

### Subjects

Experiments were conducted in the Department of Biomedical Sciences within the Joan C. Edwards School of Medicine at Marshall University and were approved by the Institutional Animal Care and Use Committee at Marshall University. Experiments were conducted in accordance with the National Institutes of Health Guidelines for the Care and Use of Laboratory Animals and with the ARRIVE guidelines. Efforts were made to reduce the number of animals used and to minimize animal pain and discomfort. Female C57BL/6J and DBA/2J mice were ordered from The Jackson Laboratory (JAX) at 3 weeks of age and were used as experimental subjects (JAX stock numbers 000664 and 000671, respectively).

### Housing conditions

Upon arrival from JAX, mice were assigned individual identification numbers. Using these numbers, mice were randomly assigned to either the isolation condition or enrichment condition using RAND() in Microsoft Excel. Mice in the isolation condition were housed individually in clear polycarbonate standard size mouse cages with no enrichment items. Mice in the enrichment condition were group-housed (2–6 mice per cage) in clear polycarbonate standard size rat cages that contained the following enrichment items: two vertical running wheels, a single horizontal running wheel the base of which served as a nesting box, a polycarbonate tube suspended from the cage lid, and several Nestlets. Mice were housed in these conditions for 10 weeks prior to behavioral testing. Mice remained housed in isolation or enrichment conditions throughout the study apart from the brief time that they were in the testing apparatus and several days immediately following jugular catheterization when all mice were individually housed in a standard size mouse cage to facilitate surgical recovery; Nestlets and a Shepherd Shack were provided to mice in the enrichment condition when they were individually housed during surgical recovery. Mice were maintained in a temperature-controlled environment (21 ± 1 °C) on a 12:12 light:dark cycle (lights on at 0600). Mice had free access to food and water throughout the experiment except for the brief time in the testing apparatus.

### Apparatus

Intravenous cocaine and sensory self-administration data were collected using 32 modular mouse operant conditioning chambers enclosed in sound attenuating cubicles (Med Associates; St. Albans, Vermont). The floor of each chamber consisted of bars which were covered by a single piece of white PVC to facilitate cleaning and mouse ambulation. Two retractable response levers were mounted to the left and right sides of the front wall (henceforth inactive lever and cocaine lever, respectively). A third retractable response lever (henceforth sensory lever) was mounted on the back wall directly across from the inactive lever. A stimulus light was mounted directly above each of the three levers. A house light was centrally mounted on the front wall of each chamber. A 25-gauge single-channel plastic swivel was mounted to a counterbalanced lever-arm attached to the lid of the chamber. An infusion pump was mounted within the sound attenuating cubicle outside of the operant conditioning chamber. Tubing was used to connect a 20 mL syringe mounted in the infusion pump to the swivel. During cocaine self-administration testing, tubing was used to connect the externalized catheter port on the midscapular region of the mouse to the plastic swivel. Operant conditioning chambers were controlled by two Med Associates control units using MED-PC V software.

### Sensory stimulus self-administration

Following 10 weeks of differential housing, mice were tested for 21 sessions on a stage during which they could self-administer sensory stimuli on a fixed ratio one (FR-1) schedule (henceforth sensory stage). A single session on an extinction schedule (i.e., no rewards delivered) followed the 21 FR-1 sessions. For all operant conditioning sessions at all stages in this study, session duration was 120 min, and testing occurred once per day at the same time every day seven days per week. Testing occurred during the day and mice were maintained on a standard light–dark cycle (i.e., lights on during the day and off at night).

Sessions during the sensory stage began with the illumination of the house light, extension of the sensory lever, and extension of the inactive lever. The cocaine lever remained retracted during all sessions on the sensory stage. Depressing the sensory lever resulted in the delivery of a sensory reward composed of visual, auditory, and tactile components. Depressing the inactive lever had no consequence. All lever presses were recorded. To provide the visual component of the sensory reward, the house light was extinguished, and the stimulus lights above the sensory lever and inactive lever were rapidly flashed on and off. Flash duration (1, 2, 4, or 8 s) and frequency (5, 2.5, 1.25, or 0.625 Hz) were randomized independently for each reward. The house light was re-illuminated once the flashing of the stimulus lights had ended. The auditory and tactile components of the sensory reward were accomplished by retraction of both the sensory lever and inactive lever. Lever retraction occurred as the stimulus lights began flashing, and lever extension occurred almost immediately (400 ms) following retraction. The extinction session was identical to FR-1 sessions with the exception that no sensory rewards were delivered.

We chose to use a compound reinforcer with varying flash rate and duration because we and others have found that reinforcement value of a sensory stimulus is dependent on stimulus complexity^5,24^. Specifically, the more complex the stimulus the higher the reinforcement value. Novelty may be one of the underlying drivers of this effect and may have influenced reinforcement value of the sensory stimuli in the present study. In the context of the present study, our goal was to use the sensory stimulus with the highest reinforcement value. Nevertheless, studying reinforcement from stimuli that are typically used in drug self-administration studies (i.e., static stimulus light, sound of the infusion pump, and saline infusion) may help to inform the underlying drivers of drug self-administration.

### Jugular catheterization surgery

Following the sensory stage, an indwelling catheter was implanted into the right external jugular vein under oxygen/isoflurane anesthesia using our previously described procedures^7,22,23^. Briefly, the catheter was inserted 12 mm into the jugular vein and anchored with sutures. The catheter was tunneled subcutaneously to an incision in the mid-scapular region where it was connected to an externalized catheter access port. Immediately following surgery, isolation mice were returned to their isolation cage; enriched mice were singly housed for several days in a standard size mouse cage to facilitate recovery. Enriched mice were returned to their original enrichment cage with the same cage mates as soon as they had fully recovered from surgery and at least 5 days before the first session of intravenous cocaine self-administration. During recovery, catheters were locked with a heparin solution (100 U/mL heparin/saline) which was replaced every three days. At the conclusion of the study, catheters were tested for patency with an infusion (1 µL/g) of a methohexital/saline solution (5 mg/kg). Rapid loss of muscle tone was interpreted as an indication of patency.

### Intravenous cocaine self-administration

Following surgical recovery, mice were tested on a stage during which they could intravenously self-administer cocaine (henceforth cocaine stage). The cocaine stage was identical to the sensory stage with the following exceptions: the sensory lever remained retracted during all sessions on the cocaine stage. The cocaine lever and inactive lever were extended at the beginning of the two-hour session and remained extended throughout the session. When the cocaine lever was depressed, an infusion of cocaine in a saline vehicle was delivered to the mouse (~ 20 µL per infusion; ~ 2.5 s infusion duration). Infusion duration was programmatically adjusted based on mouse weight to hold mg/kg/infusion of cocaine constant. Mice were tested on five distinct cocaine doses presented in descending order: 1.0, 0.32, 0.1, 0.056, 0.032 mg/kg/infusion. Mice were tested for at least five sessions on the 1.0 mg/kg/infusion dose and at least two sessions on each of the other four doses. The stimulus light above the cocaine lever was turned on at infusion onset and turned off after five seconds. To reduce the chance of overdose, a 25 s timeout began at infusion onset. During the timeout, lever presses were recorded but had no consequence. The house light was turned off at the beginning of the infusion and turned back on at the end of the timeout. Importantly, during sessions on the cocaine stage, levers were never retracted and neither the house light nor any stimulus lights were flashed.

To maintain patency, catheters were flushed before and after each daily testing session with 20 µL of a heparin lock solution (100 U/mL heparin/saline). To forestall bacterial infection, mice were infused (2 µL/g) with an enrofloxacin/saline solution (22.7 mg/kg) immediately before the heparin flush at the end of each session. Cocaine hydrochloride (CAS Registry Number: 53–21-4) was obtained from the NIDA Drug Supply Program. Cocaine doses were calculated as the salt. All drugs were dissolved in 0.9% USP sterile saline. All solutions were filtered through 0.22 µm syringe filters.

### Reward choice

Between the 1.0 and 0.32 mg/kg/infusion doses on the cocaine stage, mice were tested on a stage during which they could self-administer both sensory stimuli and a 1.0 mg/kg/infusion dose of cocaine (henceforth reward choice stage). Mice were tested for five sessions on the reward choice stage which was identical to the sensory stage and cocaine stage with the exception that both rewards were available. Specifically, all three levers in the chamber were extended (i.e., sensory lever, cocaine lever, inactive lever) and mice could self-administer sensory rewards and cocaine rewards. As in the sensory stage, sensory reward duration was 1, 2, 4, or 8 s; neither sensory rewards nor cocaine rewards were available during that time. As in the cocaine stage, the timeout following a cocaine reward was 25 s; neither cocaine rewards nor sensory rewards were available during that time. With those two exceptions, both cocaine rewards and sensory rewards were always available during the 120-min sessions.

Following collection of all data presented in this manuscript, some mice were tested on additional cocaine self-administration stages including extinction and reinstatement. Those data are not presented here because insufficient data were collected for statistical analysis.

Prior to the reward choice stage described in this section, mice were tested for 21 sessions on the sensory stage whereas they were tested for 5 sessions on the intravenous cocaine self-administration stage at the 1.0 mg/kg/infusion dose (both stages described above). Regarding the design decision to use a different number of sessions on the sensory stage relative to the cocaine stage, we reasoned that following acquisition of the lever pressing response on the sensory stage, experimental requirements on subsequent stages would be less challenging and therefore would be accomplished in fewer sessions. Specifically, on the sensory stage mice were required to acquire a novel lever pressing response, whereas on the cocaine stage mice were required to adapt that response to a different lever and a different reward. It is possible that the greater training with a sensory reward may have reduced the value of that reward during the choice stage via habituation to novelty.

### Statistical methods

We used ANOVA to test the effects of independent variables on dependent variables. Independent variables were strain, housing, lever, stage, session, and time bin. Dependent variables were lever presses and preference for the cocaine lever relative to the sensory lever (calculated as a percentage). We assessed normality of measures using normal probability plots. We assessed homogeneity of variance using Mauchly’s test of sphericity. We used the Huynh–Feldt correction to address violations of homogeneity of variance. We used Fisher's Least Significant Difference procedure to perform multiple comparisons.

## Results

### Attrition

**Figure 1:**
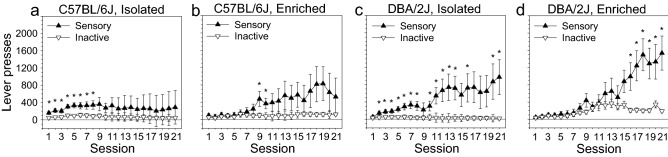
Acquisition of sensory stimulus self-administration. (**a**–**d**) Acquisition curves for sensory stimulus self-administration for each of the experimental groups in the study.

**Figure 2:**
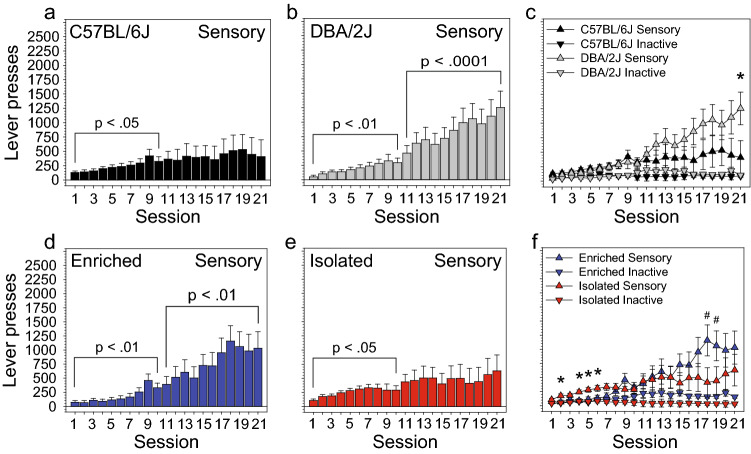
Influence of strain and housing condition on sensory stimulus self-administration. (**a**,**b**) Lever pressing across sessions was influenced by strain [Session × Strain: F (20, 1760) = 2.98, p < 0.05]. Sensory lever pressing increased significantly across the first half of the sensory stage (session 1 vs 10) for both DBA/2J mice (p < 0.01) and C57BL/6J mice (p < 0.05). Sensory lever pressing of DBA/2J mice, but not C57BL/6J mice, continued to increase during the second half of the sensory stage (session 11 vs 21: p < 0.0001). **(c)** Consequently, by the final session, sensory but not inactive lever pressing of DBA/2J mice was significantly greater than that of C57BL/6J mice (p < 0.05). (**d**,**e**) Lever pressing across sessions was also influenced by housing condition [Session × Housing: F (20, 1760) = 3.18, p < 0.05]. Sensory lever pressing increased significantly across the first half of the sensory stage (session 1 vs 10) for both enriched mice (p < 0.01) and isolated mice (p < 0.05). Sensory lever pressing of enriched mice, but not isolated mice, continued to increase during the second half of the sensory stage (session 11 vs 21: p < 0.01). (**f**) Consequently, sensory lever pressing of enriched mice was greater than that of isolated mice during the second half of the sensory stage, and this difference approached statistical significance on several sessions. Sensory lever pressing of isolated mice was significantly greater than that of enriched mice on several sessions during the first half of the sensory stage. *p < 0.05; ^#^p < 0.10.

**Figure 3:**
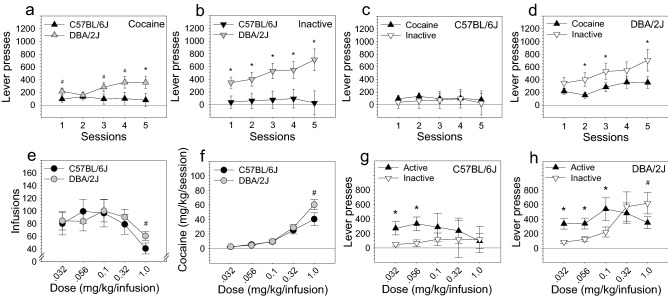
Influence of strain and dose on intravenous cocaine self-administration. (**a**,**b**) Following the sensory stage, mice were tested on the intravenous cocaine self-administration stage for five sessions on the 1.0 mg/kg/infusion dose prior to being tested on the remaining doses. On these five sessions, ANOVA revealed that DBA/2J mice pressed the cocaine lever and inactive lever significantly more than C57BL/6J mice [Strain × Lever: F (1, 35) = 7.44, p < 0.01]. (**c**,**d**) Cocaine and inactive lever pressing of C57BL/6J mice was equivalent during these five sessions, whereas DBA/2J mice pressed the inactive lever significantly more than the active lever. (**e**,**f**) Cocaine dose significantly influenced number of infusions on the dose–response curve [Dose: F (4, 116) = 6.63, p < 0.001] and cocaine intake on the dose–response curve [Dose: F (4, 116) = 61.41, p < 0.001]. (**g**,**h**) Lever pressing on the dose–response curve was significantly influenced by cocaine dose [Lever × Dose: F (4, 116) = 3.82, p < 0.05]. ANOVA did not reveal a statistically significant effect of housing on cocaine self-administration in C57BL/6J and DBA/2J mice. *p < 0.05; ^#^p < 0.10.

**Figure 4:**
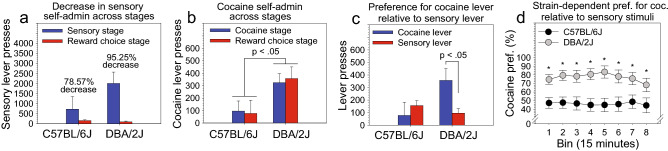
Influence of strain on the preference for intravenous cocaine relative to sensory stimuli. (**a**) Sensory lever presses were significantly lower on the reward choice stage relative to the sensory stage [Stage: F (1, 35) = 9.24, p < 0.01]. The effect of stage was much larger in DBA/2J mice (p < 0.01) relative to C57BL/6J mice (p = 0.35). (**b**) To determine if intravenous cocaine self-administration was influenced by the availability of sensory stimuli, we compared cocaine lever presses on the cocaine dose–response stage to cocaine lever presses on the reward choice stage (1.0 mg/kg/infusion for both stages). Strain, but not stage, significantly influenced cocaine lever presses [Strain: F (1, 35) = 4.81, p < 0.05]. (**c**) On the reward choice stage, preference for the cocaine lever relative to the sensory lever varied significantly as a function of strain [Strain × Lever: F (1, 35) = 5.11, p < 0.05]. DBA/2J mice, but not C57BL/6J mice, pressed the cocaine lever significantly more than the sensory lever on the reward choice stage (p < 0.05). (**d**) When preference for cocaine relative to sensory stimuli was calculated as a percentage and examined across time bins, cocaine preference was significantly greater in DBA/2J mice (77.26%) relative to C57BL/6J mice (46.45%) [Strain: F (1, 35) = 11.75, p < 0.01]. This was true when collapsing on time bin (p < 0.01) and when considering the strain difference at each individual time bin (p < 0.05 at each 15-min bin). *p < 0.05.

**Figure 5:**
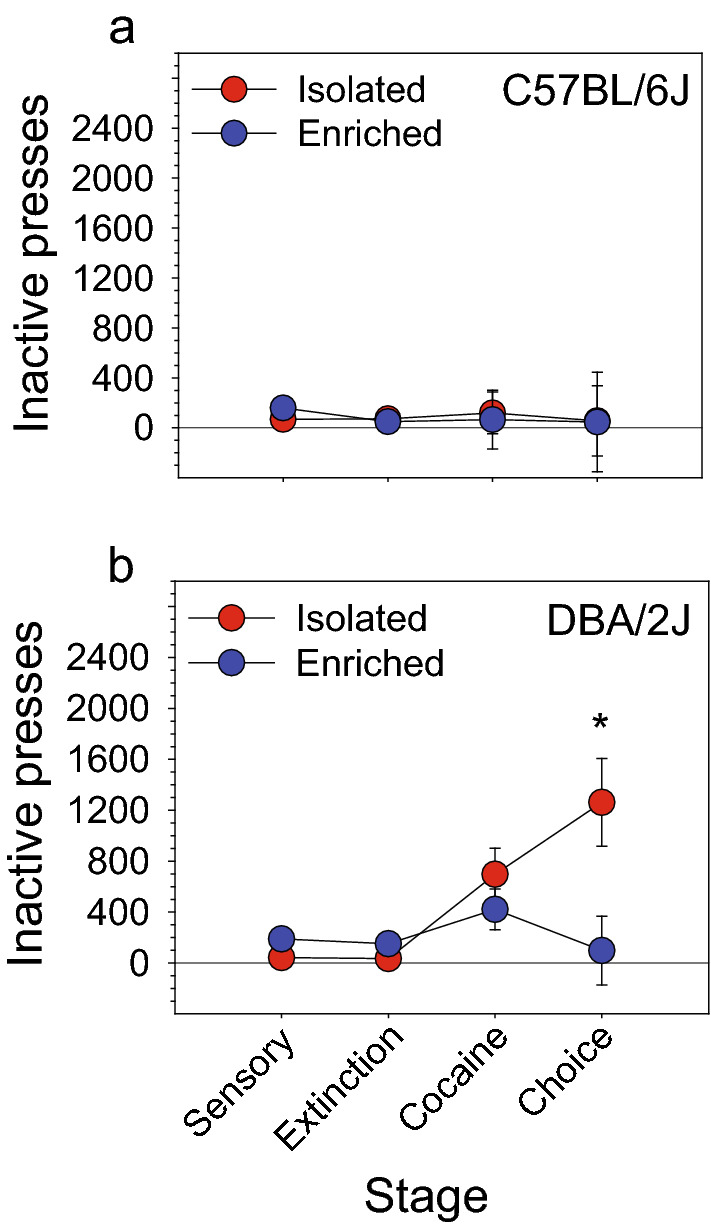
Strain-dependent effect of housing condition on inactive lever pressing (**a**,**b**) On the reward choice stage, isolated DBA/2J mice pressed the inactive lever significantly more (p < 0.05) than enriched DBA/2J mice; in contrast, inactive lever pressing of isolated and enriched C57BL/6J mice did not differ on the reward choice stage. Prior to the reward choice stage, inactive lever pressing did not differ as a function of housing in either strain. This phenomenon may reflect an isolation-induced perseverative reward seeking phenotype in DBA/2J mice.

The number of mice from the four strain/housing subgroups that completed the sensory stage, cocaine stage, and reward choice stage is shown in Table 1. Mice that completed the sensory stage (N = 93) were used in the analyses illustrated in Figs. 1, 2, and S1; one of these mice (C57BL/6J, isolated) was automatically dropped from the repeated measures analysis because data were not available for one of the sessions. Mice that completed the reward choice stage (N = 39) were used in the analyses illustrated in Figs. 3, 4, 5, and S2 with the caveat that only those mice that completed all doses on the cocaine dose–response (N = 33) were used in the dose–response analyses in Fig. 3e–h. Attrition from the sensory stage to the reward choice stage was caused by jugular vein collapse during surgery or irritation at the catheter port following surgery. Attrition following the reward choice stage was caused by loss of catheter patency. Surgical attrition was relatively high on this experiment because the surgeons were new to the intravenous jugular catheterization technique.

**Table 1:** Sample size of the experimental subgroups at each experimental stage.

Stage	Housing condition		
	Isolated	Enriched	Total
Sensory			
C57BL/6J	26	20	46
DBA/2J	23	24	47
Reward choice			
C57BL/6J	12	6	18
DBA/2J	8	13	21
Cocaine dose–response			
C57BL/6J	10	4	14
DBA/2J	7	12	19

### Sensory stimulus self-administration

Sensory self-administration data for all experimental groups is illustrated in Fig. 1. Mice exhibited a significant preference for the sensory lever relative to the inactive lever, and this effect increased across sessions [Session × Lever: F (20, 1760) = 6.54, p < 0.01] (Fig. S1). As a group, mice pressed the sensory lever significantly more than the inactive lever on all sessions (p < 0.05 on all 21 sessions).

Lever pressing across sessions was also influenced by strain [Session × Strain: F (20, 1760) = 2.98, p < 0.05]. Sensory lever pressing increased significantly across the first half of the sensory stage (session 1 vs 10) for both DBA/2J mice (p < 0.01) and C57BL/6J mice (p < 0.05) (Fig. 2a,b). Sensory lever pressing of DBA/2J mice but not C57BL/6J mice continued to increase during the second half of the sensory stage (session 11 vs 21: p < 0.0001). Consequently, by the final session, sensory but not inactive lever pressing of DBA/2J mice was significantly greater than that of C57BL/6J mice (p < 0.05) (Fig. 2c).

Lever pressing across sessions was also influenced by housing condition [Session × Housing: F (20, 1760) = 3.18, p < 0.05]. Sensory lever pressing increased significantly across the first half of the sensory stage (session 1 vs 10) for both enriched mice (p < 0.01) and isolated mice (p < 0.05) (Fig. 2d,e); sensory lever pressing of isolated mice was significantly greater than that of enriched mice on several sessions during the first half of the sensory stage (p < 0.05) (Fig. 2f). Sensory lever pressing of enriched mice, but not isolated mice, continued to increase during the second half of the sensory stage (session 11 vs 21: p < 0.01) (Fig. 2d). Consequently, sensory lever pressing of enriched mice was greater than that of isolated mice during the second half of the sensory stage, and this difference approached statistical significance on two sessions (Fig. 2f). Notably, we did not detect a statistically significant interaction of strain and housing.

Following the final FR-1 session on the sensory stage, mice were tested on a single extinction session. Sensory lever pressing on the extinction session was strongly and significantly predicted by sensory lever pressing on the final FR-1 session [*r* (90) = 0.93, p < 0.001]. ANOVA revealed a significant effect of strain [Strain: F (1, 88) = 4.40, p < 0.05] and an interaction of strain, lever, and session that approached significance [Strain × Lever × Session: F (1, 88) = 3.46, p = 0.07]. For mice as a group, we observed a significant decrease in lever pressing on the extinction session relative to the final FR-1 session [Session: F (1, 88) = 15.14, p < 0.001], and this effect varied as a function of lever [Lever × Session: F (1, 88) = 11.15, p < 0.01]. Lever pressing on both the sensory lever and inactive lever decreased significantly on the extinction session (p < 0.01 for both tests): sensory lever pressing decreased by 43.43%, and inactive lever pressing decreased by 29.31%.

Due to surgical attrition, some of the mice tested on the sensory stage were not tested on subsequent stages (Table 1). Sensory stimulus self-administration for just the mice that completed the reward choice stage is provided in Fig. S2.

### Intravenous cocaine self-administration

Following the sensory stage, mice were tested on the intravenous cocaine self-administration stage. Mice were initially tested for five sessions on the 1.0 mg/kg/infusion dose before being tested on the remaining doses that would form the dose–response curve. It should be noted that these first five sessions do not represent traditional “acquisition” of cocaine self-administration. Specifically, mice had already acquired a lever pressing response for sensory stimuli and, when cocaine became available, were then required to rapidly adapt that previously learned response to a lever in a different position in the chamber that delivered a different reinforcer (cocaine) in the absence of the prior reinforcer (sensory stimuli). Under these conditions, ANOVA revealed that DBA/2J mice pressed the cocaine lever (Fig. 3a) and inactive lever (Fig. 3b) significantly more than C57BL/6J mice [Strain × Lever: F (1, 35) = 7.44, p < 0.01]. Cocaine and inactive lever pressing of C57BL/6J mice was equivalent during these five sessions (Fig. 3c), whereas DBA/2J mice pressed the inactive lever significantly more than the cocaine lever (Fig. 3d). The elevated inactive lever pressing likely reflects sensation seeking (i.e., attempts to self-administer sensory stimuli) in DBA/2J mice which was unsurprisingly lower in C57BL/6J mice considering strain differences in sensory stimulus self-administration (Fig. 2a–c).

On the dose–response curve, cocaine dose significantly influenced number of infusions [Dose: F (4, 116) = 6.63, p < 0.001] (Fig. 3e) and cocaine intake [Dose: F (4, 116) = 61.41, p < 0.001] (Fig. 3f). For infusions, we observed both an ascending limb and a robust descending limb on the dose–response curve. On the descending limb (i.e., higher doses), the number of infusions significantly increased as cocaine dose decreased (1.0 mg/kg vs 0.1 mg/kg: p < 0.001) (Fig. 3e). On the cocaine intake curve (Fig. 3f), we observed significantly reduced cocaine intake at each dose, despite the significant increase in infusions as dose was reduced (p < 0.001 for each dose relative to all other doses). For cocaine intake, the interaction of strain and dose approached statistical significance [Strain × Dose: F (4, 116) = 2.85, p = 0.07]. This effect was predominantly driven by strain differences at the highest cocaine dose. Specifically, on the 1.0 mg/kg/infusion dose, number of infusions and cocaine intake were both higher for DBA/2J relative to C57BL/6J mice, and this difference approached statistical significance (p = 0.08 for both tests).

For mice as a group, lever pressing on the dose–response curve was significantly influenced by cocaine dose [Lever × Dose: F (4, 116) = 3.82, p < 0.05], and the interaction of strain and cocaine dose approached statistical significance [Strain × Dose: F (4, 116) = 2.40, p = 0.08]. Both C57BL/6J mice and DBA/2J mice pressed the cocaine lever significantly more than the inactive lever on the lowest two cocaine doses (p < 0.05 for all comparisons) (Fig. 3g,h); DBA/2J mice also pressed the cocaine lever significantly more than the inactive lever on the 0.1 mg/kg dose (p < 0.05). DBA/2J mice pressed the inactive lever more than the cocaine lever on the highest cocaine dose, and this difference approached statistical significance (p = 0.08). ANOVA did not reveal a statistically significant effect of housing on cocaine self-administration in C57BL/6J and DBA/2J mice.

### Preference for intravenous cocaine relative to sensory stimuli

To determine if sensory stimulus self-administration changed across experimental stages, we compared sensory lever presses on the sensory stage (mean of final three sessions) to sensory lever presses on the reward choice stage (mean of final three sessions) (Fig. 4a). ANOVA revealed that stage significantly influenced sensory lever presses [Stage: F (1, 35) = 9.24, p < 0.01] such that presses were significantly lower on the reward choice stage relative to the sensory stage (p < 0.01). Notably, the effect of stage was much larger in DBA/2J mice (p < 0.01) relative to C57BL/6J mice (p = 0.35). Specifically, on the reward choice stage, DBA/2J mice decreased their sensory lever pressing by 95.25% relative to the sensory stage (95.32 vs 2006.44), whereas C57BL/6J mice decreased their sensory lever pressing by 78.57% (155.60 vs 726.17). ANOVA did not reveal a statistically significant influence of housing condition on this effect or any other effect described in this section.

To determine if intravenous cocaine self-administration was influenced by the availability of sensory stimuli, we compared cocaine lever presses on the cocaine stage (1.0 mg/kg/infusion; mean of final three sessions) to cocaine lever presses on the reward choice stage (1.0 mg/kg/infusion; mean of final three sessions). ANOVA did not reveal a statistically significant effect of stage on cocaine lever presses. However, strain significantly influenced cocaine lever presses [Strain: F (1, 35) = 4.81, p < 0.05] such that DBA/2J mice intravenously self-administered significantly more cocaine relative to C57BL/6J mice (p < 0.05) (Fig. 4b). Because of attrition due to a loss of catheter patency across dose–response testing (Table 1), the sample size for the analysis in Fig. 4b (N = 39) in which a significant strain effect was observed at the 1.0 mg/kg/infusion dose was higher than that for the analysis in Fig. 3e (N = 33) in which the strain effect only approached statistical significance on the 1.0 mg/kg/infusion dose.

To determine if cocaine lever presses and sensory lever presses differed significantly when both rewards were available, we compared cocaine lever presses on the reward choice stage (1.0 mg/kg/infusion; mean of final three sessions) to sensory lever presses on the reward choice stage (mean of final three sessions). ANOVA revealed that preference for the cocaine lever relative to the sensory lever varied significantly as a function of strain [Strain × Lever: F (1, 35) = 5.11, p < 0.05]. Post hoc tests indicated that DBA/2J mice, but not C57BL/6J mice, pressed the cocaine lever significantly more than the sensory lever on the reward choice stage (p < 0.05) (Fig. 4c).

To directly compare reward preference of C57BL/6J and DBA/2J mice, we performed the same ANOVA using percentage presses on the cocaine lever relative to the sensory lever as the dependent variable. We also added session time (15-min bins) as an independent variable to determine if preference varied across the session. ANOVA revealed a significant main effect of strain [Strain: F (1, 35) = 11.75, p < 0.01] on preference for cocaine relative to sensory stimuli (Fig. 4d). Post hoc tests revealed that the preference for cocaine relative to sensory stimuli was significantly greater in DBA/2J mice (77.26%) relative to C57BL/6J mice (46.45%); this was true when collapsing on time bin (p < 0.01) and when considering the strain difference at each individual time bin (p < 0.05 at each 15-min bin).

### Strain-dependent effect of housing condition on inactive lever pressing

In the context of performing the analysis of cocaine seeking and sensory stimulus seeking during the choice stage, we identified an effect of housing on inactive lever pressing that was specific to the DBA/2J mouse strain. ANOVA revealed a statistically significant two-way interaction of stage and housing [Stage × Housing: F (3, 105) = 3.44, p < 0.05] and a main effect of strain [Strain: F (1, 35) = 4.97, p < 0.05]. The three-way interaction of strain, stage, and housing was marginally significant [Strain × Stage × Housing: F (3, 105) = 3.00, p = 0.07]. Post hoc tests revealed that inactive lever pressing among these groups was statistically equivalent at all stages prior to the reward choice stage. However, on the reward choice stage, isolated DBA/2J mice pressed the inactive lever significantly more (p < 0.05) than enriched DBA/2J mice (Fig. 5b). Inactive lever pressing of isolated and enriched C57BL/6J mice did not differ (Fig. 5a).

## Discussion

### Summary

In the present study, we used the two founder strains of the BXD recombinant inbred mouse panel to develop a novel behavioral assay of addiction vulnerability. Our goal was to leverage the construct validity of the gold-standard intravenous drug self-administration paradigm to quantify the preference for intravenous cocaine self-administration relative to sensory stimulus self-administration. Because complex sensory stimuli are natural rewards in both mice and humans^2–6^ and are key components of activities that humans find rewarding, we reasoned that a strong preference for an intravenous drug reward relative to a sensory reward would serve as an endophenotype of addiction vulnerability, whereas a strong preference for a sensory reward relative to a drug reward would serve as an endophenotype of addiction resistance. In the context of developing this assay, we replicated our previous findings of strain effects (Fig. 2a–c) and housing effects (Fig. 2d–f) on sensory stimulus self-administration and identified strain effects on cocaine self-administration (Figs. 3, 4b). Most importantly, we identified a robust and statistically significant preference for cocaine relative to sensory stimuli in DBA/2J mice, but not C57BL/6J mice, (Fig. 4c,d) and a distinct pattern of inactive lever pressing in isolated DBA/2J mice relative to other mice (Fig. 5). The strain differences observed in the present study indicate that the full panel of BXD mouse strains can be used to identify the genetic mechanisms driving the preference for an addictive drug relative to a natural reward.

### Strain difference in the preference for cocaine relative to sensory stimuli in BXD founders: implications for systems genetics dissection using the full BXD panel

The most important finding from this study was the robust strain difference in the preference for cocaine relative to sensory stimuli. Specifically, when DBA/2J mice were provided simultaneous access to (1) a lever that would deliver a cocaine infusion and (2) a separate lever that would deliver a sensory reward, they exhibited a strong and significant preference for the cocaine lever (77.26%) (Fig. 4c,d). In contrast, C57BL/6J mice exhibited no significant preference for the cocaine lever relative to the sensory lever (46.45%).

The significantly greater preference for cocaine relative to sensory stimuli in DBA/2J mice relative to C57BL/6J mice was driven by two factors. First, during both the cocaine stage and the reward choice stage (Fig. 4b), DBA/2J mice self-administered significantly more cocaine than C57BL/6J mice. Notably, cocaine self-administration neither increased nor decreased from the cocaine stage to the reward choice stage in either strain. Second, although both C57BL/6J and DBA/2J mice reduced sensory lever pressing when cocaine became available during the reward choice stage, this reduction was much greater in DBA/2J mice (Fig. 4a). Indeed, the difference in reduction between the two strains was such that although DBA/2J mice self-administered significantly *more* sensory stimuli relative to C57BL/6J mice on the sensory stage, they self-administered *fewer* sensory stimuli relative to C57BL/6J mice on the reward choice stage (Figs. 2a–c, 4a). Collectively, in DBA/2J mice relative to C57BL/6J mice, the greater baseline cocaine self-administration and the greater reduction in sensory stimulus self-administration when cocaine was introduced during the reward choice stage resulted in a significantly greater preference for cocaine relative to sensory stimuli on the reward choice stage.

It should be noted that some of the reduction in sensory lever pressing (Fig. 4a) may have been caused by tethering. Specifically, mice were not tethered during the sensory stage because that stage occurred prior to jugular catheterization; in contrast, mice were tethered during the reward choice stage. Consequently, the greater reduction in sensory lever pressing in DBA/2J mice relative to C57BL/6J mice could have resulted from strain differences in the response to tethering. In future studies, this question could be resolved by taking a second baseline of sensory lever pressing when mice are tethered following surgery or performing surgery prior to sensory stimulus self-administration and tethering mice throughout the experiment. It is also possible that strain dependent extinction of sensory stimulus self-administration influenced subsequent responding during the choice stage. In future studies, this could be addressed by excluding the extinction stage.

Despite some uncertainty about the underlying behavioral drivers, the difference in the preference for cocaine relative to sensory stimuli between C57BL/6J and DBA/2J mice was robust and statistically significant (Fig. 4c,d). Because C57BL/6J and DBA/2J mice are the founder strains of the BXD recombinant inbred panel, these findings reveal that the full BXD panel can be used to dissect the genetic mechanisms underlying the preference for an addictive drug relative to a natural reward. Moreover, in future studies, we could more fully characterize these genetic mechanisms by using additional operant conditioning schedules (e.g., progressive ratio, fixed ratio greater than 1) to experimentally manipulate the effort required to receive a reward.

### Effects of strain and housing condition on inactive lever pressing

During the process of characterizing the preference for a cocaine reward relative to a sensory reward, we identified a strain-by-housing interaction influencing inactive lever pressing. Specifically, during the reward choice stage, isolated DBA/2J mice exhibited significantly greater levels of inactive lever pressing relative to environmentally enriched DBA/2J mice (Fig. 5b); this housing effect was completely absent in C57BL/6J mice (Fig. 5a). This is a curious finding because the psychological construct being manifested is not immediately clear. One possibility is that the increased inactive lever pressing in isolated DBA/2J mice reflects perseveration of the sensation seeking response. Specifically, DBA/2J mice, but not C57BL/6J mice, increased inactive lever pressing on the cocaine stage when sensory rewards were no longer available (Figs. 3b, 5); notably, this effect was not influenced by housing. When sensory stimuli again became available during the reward choice stage, environmentally enriched DBA/2J mice *decreased* inactive lever pressing whereas isolated DBA/2J mice continued to *increase* inactive lever pressing. This pattern suggests that DBA/2J mice in both housing conditions were seeking out sensory stimuli (by pressing the inactive lever) when those stimuli became unavailable during the cocaine stage. When sensory stimuli again became available during the reward choice stage, isolated DBA/2J mice persisted in this ineffective strategy whereas enriched DBA/2J mice did not. This phenomenon may reflect an isolation-induced perseverative reward seeking phenotype in DBA/2J mice.

Although we did not identify statistically significant housing effects on cocaine self-administration or preference for cocaine relative to sensory stimuli, it is possible that undetected housing effects influencing cocaine self-administration or the preference for cocaine relative to sensory stimuli exist in mice and would have been detected with a larger sample size using the BXD founders or with two mouse strains exhibiting greater vulnerability and resistance to the housing effect (e.g., strains from the BXD or Collaborative Cross recombinant inbred panels).

### Effects of strain on sensory stimulus self-administration

During the sensory stage, DBA/2J mice self-administered significantly more sensory stimuli than C57BL/6J mice (Fig. 2a–c). These results confirm strain differences in sensory stimulus self-administration identified in two of our previous studies^5,6^ and support the hypothesis that the homeostatic set point of sensory stimulation is heritable. This hypothesis posits that organisms have an innate preference for a specific level of sensory stimulation and will behave in ways which maintain that optimum level^25^. Studies in humans^3,26^ and mice^4,27^ suggest that this setpoint, as indexed through level of sensory stimulus self-administration, covaries with addiction-like behaviors and is driven by reward system circuitry. Findings from the present study support these conclusions: relative to C57BL/6J mice, DBA/2J mice self-administered more sensory stimuli and, when both sensory stimuli and cocaine were available, exhibited a preference for cocaine. A systems genetics study using the full BXD panel would enable a direct test of the hypothesis that this relationship is heritable. Specifically, the observation of a positive genetic correlation between sensory stimulus self-administration and preference for intravenous cocaine relative to sensory stimuli would indicate that these phenomena are driven by shared genetic mechanisms. These behavioral data could then be integrated with genetic and transcriptomic data to dissect the mechanisms underlying the observed relationship.

### Effects of housing condition on sensory stimulus self-administration

During the sensory stage, self-administration of environmentally enriched mice differed in two ways relative to that of isolation housed mice (Fig. 2d–f). First, isolation housed mice self-administered significantly more sensory stimuli during early FR-1 sessions when the number of sensory lever presses was low relative to later sessions. Second, environmentally enriched mice self-administered more sensory stimuli during later FR-1 sessions when the number of sensory lever presses was relatively high. Regarding housing difference during early FR-1 sessions, one explanation is that isolated mice were significantly more active and that this resulted in an overall increase in lever pressing. Specifically, isolation housed rodents are significantly more active in a novel open field relative to enriched or standard housed rodents^28–30^; we and others have found this to be true in BXD founder strains^21,31^. It is also possible that isolation housing caused an increase in the sensory stimulus setpoint resulting in higher sensory stimulus self-administration; this conclusion is consistent with findings from a similar study using rats^32^.

In our previous study^6^, DBA/2J mice that were isolation housed exhibited significantly more robust sensory lever pressing during the extinction stage relative to enriched DBA/2J mice; this phenomenon was not observed in C57BL/6J mice. Notably, this effect in DBA/2J mice was quite robust, and it was observed in both male and female mice. In the present study, although we observed a statistically significant effect of strain during the extinction session on the sensory stage, we did not observe a statistically significant effect of housing condition or interaction of housing condition and strain. One notable difference between these two studies is that, in the present study, FR-1 sessions preceded the extinction stage; in contrast, in the previous study, progressive ratio sessions preceded the extinction stage. This may be relevant because the progressive ratio schedule shapes mice to persist in responding despite infrequent reward delivery and, consequently, results in dramatic changes in response characteristics^33^. Thus, strain dependent effects of housing condition on extinction responding may be dependent on prior operant conditioning schedules.

### Notes on experimental design and methodology

#### Acquisition criteria

In the present study, mice acquired a lever pressing response for sensory stimuli (Figs. 1, 2). Following this, mice acquired a lever pressing response for a cocaine infusion (Fig. 3). One of the challenges with this approach is the difficulty in establishing acquisition of the lever pressing response for cocaine following acquisition of the lever pressing response for sensory stimuli. The reason for this is that once mice begin pressing a lever for a reward, they will continue pressing levers during extinction conditions. We attempted to address this issue in the present study by using distinct levers for delivery of sensory stimuli and cocaine infusions. Thus, during the cocaine stage, a preference for the cocaine lever relative to the inactive lever would indicate volitional cocaine seeking. In this regard, the dose–response curve data support that acquisition of cocaine self-administration did indeed occur for both C57BL/6J and DBA/2J strains. Specifically, we observed a significant effect of lever, and both strains pressed the cocaine lever significantly more than the inactive lever on lower doses (Fig. 3g,h). A preference for the cocaine lever was not present on higher doses, and there are a number of possible explanations for this phenomenon. First, at higher doses, cocaine satiation may have been reached before baseline lever pressing was reached; this could have resulted in equivalent cocaine and inactive lever pressing despite volitional cocaine seeking. Second, volitional cocaine seeking on the cocaine lever may have occurred simultaneously with sensory stimulus seeking on the inactive lever; this could have resulted in significantly higher inactive lever pressing relative to cocaine lever pressing.

#### Additional control groups

As discussed above, the inactive lever served as the primary control in the present study. An additional method of control would be to add a separate group that could lever press but was exposed to neither sensory stimuli nor cocaine. This would establish baseline lever pressing to which lever pressing in the rewarded group could be compared. Moreover, a group that was exposed to sensory stimuli and cocaine in a non-contingent fashion would allow dissection of the direct effect of these rewards.

#### Possible improvements to the choice procedure

One of the limitations of the present study is that we used an FR-1 schedule during the choice stage. Consequently, when choosing between cocaine and sensory stimuli, it may have been possible for mice to self-administer to the point of satiety for both rewards. To address this issue in future studies, the design could be adapted such that access to one reward is reduced if the other reward is chosen. This could be accomplished by including a punishment component to the task, limiting the total number of available rewards during a session, or incrementally increasing the amount of work required to receive a reward (e.g., progressive ratio schedule).

#### Statistical power

Like many intravenous drug self-administration experiments using mice, the present study used a modest sample size. Consequently, some of the statistical tests, in particular those assessing higher order interactions, were underpowered. Therefore, the absence of a statistically significant effect in the present study should not be interpreted as the absence of a relationship between those variables in the population.

### Conclusion

Preference for a drug reward relative to a sensory reward may be an endophenotype of addiction vulnerability. In the present study, we developed a novel behavioral assay to quantify the preference for intravenous drug self-administration relative to sensory stimulus self-administration. When a single reward was available (sensory stimuli or cocaine; delivered using distinct levers), DBA/2J mice self-administered significantly more rewards than C57BL/6J mice. When both rewards were available, DBA/2J mice exhibited a significant preference for cocaine relative to sensory stimuli; in contrast, C57BL/6J mice exhibited no preference. Relative to other groups, isolated DBA/2J mice exhibited a distinct pattern of inactive lever pressing when both rewards were available. Environmentally enriched mice exhibited a distinct pattern of sensory self-administration relative to isolation housed mice. Collectively, these data reveal strain effects, housing effects, or both on reward self-administration and preference. Most importantly, this study reveals that genetic mechanisms underlying preference for a drug reward relative to a nondrug reward can be dissected using the full BXD panel.

## Supplementary Information

Supplementary Figures.

## Data Availability

The dataset used in this study is available from the corresponding author on request.
