# Supplementary material for: Effects of isolation housing stress and mouse strain on intravenous cocaine self-administration, sensory stimulus self-administration, and reward preference
Source: Sci Rep. 2023 Feb 16;13:2810. doi: 10.1038/s41598-023-29579-9 (PMC9935522; doi:10.1038/s41598-023-29579-9)
Supplement: Supplementary file 1 — Supplementary Figures. [file 41598_2023_29579_MOESM1_ESM.pdf]

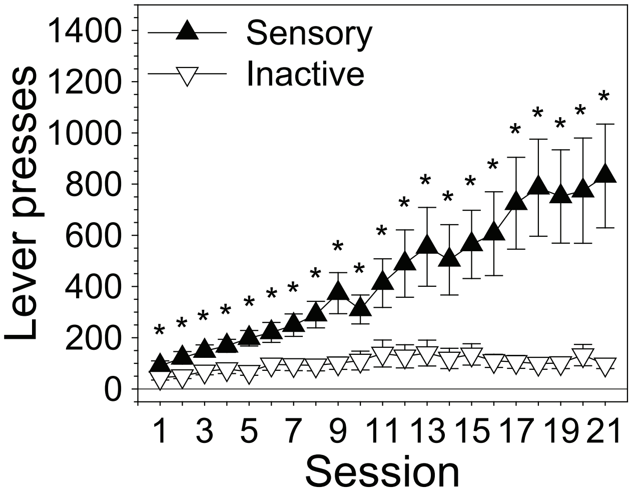

Figure S1. Acquisition of sensory stimulus self-administration in mice that completed the sensory stage (see Table 1). As a group, mice exhibited a significant preference for the sensory lever relative to the inactive lever, and this effect increased across sessions [Session x Lever: $F(20, 1760) = 6.54$, $p < .01$]. Mice pressed the sensory lever significantly more than the inactive lever on all sessions ($p < .05$ on all 21 sessions).

* $p < .05$

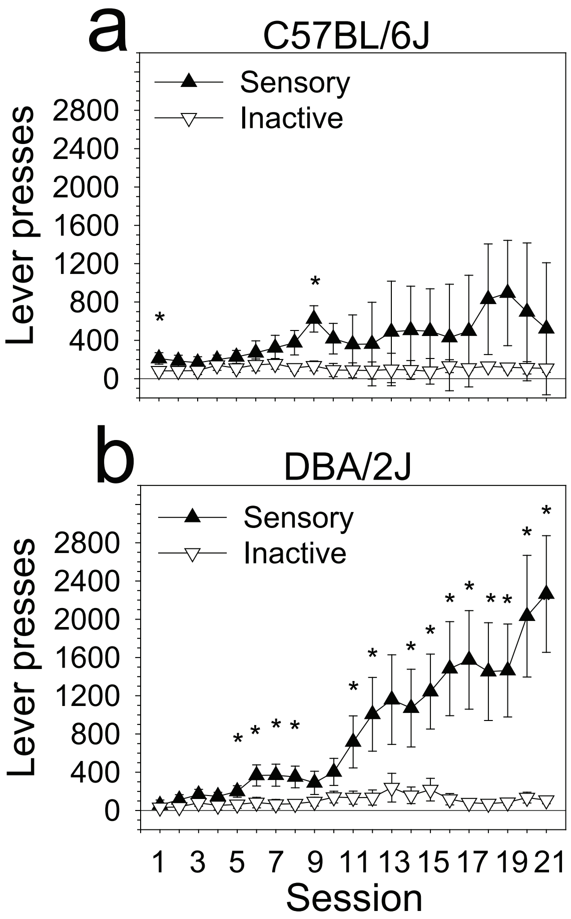

Figure S2. Acquisition of sensory stimulus self-administration in C57BL/6J and DBA/2J mice that completed the reward choice stage (see Table 1). As a group, mice exhibited a significant preference for the sensory lever relative to the inactive lever [Lever: $F(1, 34) = 7.29$, $p < .05$], and this effect increased across sessions [Session x Lever: $F(20, 680) = 4.52$, $p < .05$]. In this sample, strain approached significance [Strain x Session: $F(20, 680) = 2.32$, $p = .10$] and housing was not significant. This was likely due to the reduced sample size caused by surgical attrition and the resulting reduction in statistical power.

$p < .05$
